# Investigation of the effects of bovine collagen peptides and mixed berries on rheological properties and biological activity of egg white-based beverage *via* central composite design

**DOI:** 10.3389/fnut.2022.1011553

**Published:** 2023-02-09

**Authors:** Adrienn Varga-Tóth, Csaba Németh, István Dalmadi, Tamás Csurka, Renáta Csorba, Majd Elayan, Munkhnasan Enkhbold, Karina Hidas, László Ferenc Friedrich

**Affiliations:** ^1^Department of Livestocks Products and Food Preservation Technology, Institute of Food Science and Technology, Hungarian University of Agriculture and Life Sciences, Budapest, Hungary; ^2^Capriovus Ltd., Szigetcsép, Hungary

**Keywords:** egg white, dairy replacement, functional food, central composite design - response surface methodology, bioactive compounds, bovine collagen peptides, berries and fruits, rheological properties

## Abstract

Modern consumer expectations have become highly diversified: they want more opportunities to meet diverse family needs (diversity of family members in age, gender, physical activity, etc. ,) and individual health goals with a huge variety of sensorial preferences. Our research is aimed to develop a protein-dense, highly bioactive, lactose- and whey protein-free beverage applying a central composite rotational design (CCRD) with 2 factors. For this purpose, an egg white-based beverage was flavored with mixed berries (factor A) and enriched with bovine collagen peptides (factor B). After suitable sample preparation, the rheological properties were investigated by an Anton Paar MCR 92 rheometer (with CC 27 system, and flow behavior was analyzed with a Herschel-Bulkley (H-B) model). The antioxidant capacity of samples was investigated by Ferric Reducing Antioxidant Power (FRAP) method, the total anthocyanin content was estimated based on a spectrophotometric method, and the total phenolic content was determined by the Folin Ciocalteu method. Our results are figured on response surfaces demonstrating that both factors and their interactions show a positive correlation with the examined parameters. Based on the CCRD, all investigated parameters are significantly influenced by at least one aspect and can be adequately estimated for further product development.

## 1. Introduction

The demand for functional foods was already raised about a half-century ago when the first health and nutrition concerns were established. However, real definitions and first needs for regulation have been formulated since the 1980s when the first regulation was introduced in Japan (1). Finally, in 2006, the European Parliament and Council introduced their first regulation on nutritional and health claims [Reg. (EU) n. 1924/2006] (2). But despite this, due to the exploding demand for specific functional foods and food ingredients, there is still a continuously existing need for further definitions and regulations (3). As support for understanding our work, two widely accepted definitions are introduced in the following. As comprehensively formulated by Gur et al. (4), functional foods are natural or processed foods that contain biologically active compounds; that, in defined, effective, and non-toxic amounts, provide a clinically proven and documented health benefit utilizing specific biomarkers for the prevention, management, or treatment of chronic disease or its symptoms. According to a short definition by Santini et al. (5), functional foods are nutritional products that provide health and medical benefits, including the prevention and treatment of disease. In these aspects, the addition of antioxidant compounds such as anthocyanins, phenolic and polyphenolic compounds, or bioactive peptides may achieve a functional effect on foods. Regarding studies in the field of consumer acceptance (6), the term “functional food” has already reached the recognition of food consumers and has been becoming increasingly prominent in advertising campaigns which might be a successful tool for product introduction.

Fruits such as berries should be an important part of a healthy diet due to their content of bioactive compounds. The most consumed berries, such as blackberry, blueberry, cranberry, raspberry, and strawberry, are important sources of bioactive compounds and are consumed worldwide as fresh or processed products (7). An increasing interest is shown in literature and clinical experiments for the role of berries and their components in the modulation of oxidative stress (8), cardio-vascular symptoms (9), inflammation, and lipid metabolism (10, 11).

Antioxidants are an extremely heterogeneous ‘class' of compounds differing in chemical structures (i.e., hydrophilic, hydrophobic), distribution in nature (i.e., some are specific to vegetable species, others are generally present in food raw materials), and range of concentrations both in foods and in the human body (from nanograms to milligrams). Some classes of antioxidants, such as some vitamins, carotenoids, and polyphenols, merit specific attention not only because they are well represented in our diet but also because they are differently absorbed and metabolized and may exert diverse functions with significant impact on our health (12). They may have different actions, effectiveness against oxidative stress, and specificity (e.g., scavenging of superoxide, hydroxyl or peroxyl radicals, quenching of singlet oxygen or ferryl species, etc.) and biological action apart from the antioxidant one (e.g., modulation of functions such as detoxification, immune response, inflammation) (13).

Anthocyanins—a group of naturally occurring flavonoid heterosides in the plant kingdom—are well-known as natural colorant compounds from fruits and vegetables responsible for the red, purple, or blue color and are used as natural additives in many food products, providing a reddish, blueish color depending on the pH-value of the food product. Depending on their chemical structure, anthocyanins are generally considered heat and storage-sensitive compounds (14). For example, the major anthocyanins in strawberry are mono-glycosidi (e.g., pelargonidin-3-glycoside and pelargonidin-3-rutinoside) that undergo structural changes during storage which may lead to a deceleration (15). However, anthocyanins stability might be improved by the addition of peptides and polysaccharides (16).

Phenolic compounds such as polyphenols are reported as health-supporting natural compounds usually of plant origin. Flavonoids and non-flavonoids are the two major classes of phenolic compounds common to berries. Blueberries score extraordinarily well in rankings among polyphenol-rich berry fruits (17). Flavonoids (40%) and phenolic acids (59%) are the dominant phenolic compound fractions in them, their concentration may reach 3 mg/g during ripening (18).

Only a little scientific evidence is available on the effect of processing on polyphenol bioavailability. Some data are present in the literature on the effect of cooking on the stability and retention of some polyphenols (17), however, investigations on the absorption response are limited.

The addition of different protein sources may lead to a higher functionality of foods as well. For example, bovine collagen peptides are reported as functional compounds for skin protective and renewal effects e.g., in the healing of pressure ulcers (19). Dermal collagen is associated with skin and joint elasticity (20) which shows a close relation to collagen type I RNA expression and fibroblast growth after oral collagen peptide (CP) treatments (21).

The effect of concentration on the apparent viscosity of hydrocolloids is generally described by either an exponential or a power relationship (22, 23). Despite the huge availability as a waste by-product from meat livestock industries and a great interest in nutrition, only a few reports describe the potential use and characterization of collagen peptides, especially bovine collagen peptides (24), Currently about 28 types of collagen of different structure, amino acid composition, and biological role are reported (25). A collagen molecule consists of three left-handed helical polypeptide chains, rolled into a right-handed triple helix (c.a. 300 kDa). But the common feature of all collagens is a repeating Gly-X-Y amino-acid sequence (26, 27), where X and Y can be any amino acid, however, proline (Pro) and hydroxyproline (Pro-OH) residues are reported as the most often encountered (28). Collagen contains both polar [e.g., aspartic acid (Asp), glutamic acid (Glu), arginine (Arg), lysine (Lys)], and apolar [e.g., proline (Pro), serine (Ser), glycine (Gly)] amino acids (29). However, the native collagen helixes are rolled in a way to expose the hydrophilic segments to the solution, they might have amphiphilic character. The latter can be unleashed by denaturation and/or hydrolysis of the triple helix, using chemical or enzymatic reactions (30, 31).

Low-molecular-weight collagen peptides from fish skin have been presented as high immunomodulatory and antioxidant activity and better absorbable compounds than whole molecule collagens (32). Collagen peptide as a hydrolysate of collagen has been reported to have various beneficial effects, e.g., protecting skin aging, promoting better wound healing (33, 34), increasing muscle strength (35), reducing obesity (36), maintaining blood pressure (37), preventing atherosclerosis (38) and modifying lipid metabolism (39). Despite the health promotion of collagen peptides, a balanced and moderated—considering age, gender, physical and health state—daily protein and amino acid intake is still recommended to avoid overconsumption and related harmful effects (40). Despite a huge number of publications reporting the health benefits and bioactivity of collagen peptides, there is a lack of publications investigating the impacts of collagen peptides on techno-functional attributes. Global market for collagen peptides estimated at 564.4 million US$ in the year 2020, is projected to increase to 822.9 million US$ by 2027, growing at a CAGR of 5.5% over the investigated 7 years (41).

A further opportunity for functional food development is to exchange or reform or inhibit some compounds such as lactose or whey proteins. However, dairy products are considered highly nutritious food products, and increasing the absorption of several micronutrients (e.g., calcium and magnesium) their consumption is recommended on a daily basis. Some consumers have to replace them by consuming lactose-free and/or dairy-replacement products (42). Lactose intolerance and whey protein allergy have become today hot topics in the field of food and nutrition. In the last 10,000 years, the use of domesticated ruminants as a source of milk and dairy products has expanded until today when the dairy industry has become one of the largest sectors in the modern food industry, including the spread at the present time to countries such as China and Japan (43). About 70% of the adult world population is lactose-intolerant, due to low levels of intestinal lactase, (lactase-phlorizin hydrolase (LPH), a *β*-d-galactosidase). This may be due to the loss of intestinal lactase in adulthood, a condition transmitted by an autosomal recessive gene, which differs in humans according to race. According to the cultural-historical hypothesis, the mutation that allows the metabolization of lactose appeared about 10,000 years ago in the inhabitants of Northern Europe where mammalian milk continued in the diet after weaning, and lactase-persistent populations were genetically selected in some areas. Many intolerant individuals can tolerate low levels of lactose in their daily diet. Many products are marketed nowadays as alternatives to dairy products for lactose-intolerant individuals. However, the rules for low-lactose foods are currently not harmonized in the European Union (44). Bovine milk proteins are described as potential allergens affecting about 1–3% of adults and 3–5% of children under of the age 1 year worldwide (45). Cow's milk contains ~30–35 g/L of proteins and the class of lactoserum proteins (whey) represents 20% of total protein. *β*-lactoglobulin is the most immunogenic and abundant from whey proteins (c.a. 50%) (46).

Milk and dairy replacements (analogs) are produced usually from plant-driven raw materials worldwide satisfying the increasing demand for casein- and whey-protein-free products, although these products have generally poor nutritional quality (47). Although milk and dairy replacements are considered functional foods because they might have additional positive effects on human digestion. An excellent opportunity is to produce egg white-based dairy and milk replacement products. Avian eggs and egg white have been reported as natural functional foods (48). E.g., certain egg white-derived peptides can play a role in controlling the development of hypertension by exerting Vaso relaxing effects (due to ovokinin) (49). Hen egg white lysozyme-derived peptides showed moderate inhibitory activities against calmodulin-dependent phosphodiesterase (CaMPDE) and free-radical scavenging properties. Egg lysozyme hydrolysates have the potential as functional foods and nutraceuticals (50, 51).

The aim of this study is the development and investigation of a beverage containing highly bioactive proteins-containing beverage providing a huge concentration of bioactive compounds such as antioxidants and polyphenols. For this purpose, a central composite rotatable design (CCRD) was applied for investigating the effects of concentrations of bovine collagen peptides and mixed berries' concentration on the rheological properties and bioactive compounds of an egg white-based dairy replacement product.

## 2. Materials and methods

### 2.1. Materials and sample preparation

#### 2.1.1. Materials used for sample preparation

The egg white-based milk replacement “ToTu beverage” produced by Capriovus (Szigetcsép, Hungary) was used as the main ingredient in our samples. According to the producer, this milk replacement is made due to acidic and enzymatic reactions from liquid egg white (52). The product contains carbohydrates and fats only in traces. But at the same time, it is a great source of easy-digestible peptides and proteins (53), so its consumption may be recommended for people replacing bovine milk and dairy, dealing with diabetes or insulin resistance (54).

In every sample, erythritol (distributed by Bulkshop, Budapest, Hungary) was used as a low-calorie sugar-alcohol sweetener in a range of 15 % m/m. Regarding the literature, erythritol is concerned as a popular and highly accepted sugar alcohol sweetener (55). Furthermore from pilot experiments, it has been concluded that its sweet taste is similar to white sugar in plate and flavored ToTu beverages.

The frozen mixed berries (distributed by Lidl Budapest, Hungary; containing strawberries, raspberries, blueberries, and red currants) were used after thawing (from −18 to 10°C at room temperature) and homogenized in an electric shaker (PHILIPS HR3655/00 smoothie maker) for 2 min. The berries were used as a smoothie, or pulp containing flesh and skin, because antioxidant and phenolic compounds are reported to be present in high concentration in the skin of blueberries and red currant (56). Mixed berries were used in different concentrations (between 1.89 and 23.11 g /100 g) in samples according to the CCRD, as shown in **Table 2**.

The bovine collagen peptides were used because of their high popularity among collagen peptides (41) and their great bioactivity (51). They were purchased from and distributed by GAL SynergyTech Zrt. (Budapest, Hungary) and were added in different concentrations (between 3.52 and 0 g/100 g) to each sample regarding the CCRD presented in **Table 2**.

#### 2.1.2. Sample preparation

ToTu beverage, erythritol, mixed berries, and bovine collagen peptides were mixed in the adequate ratios given in CCRD and described above. After measurements of the ingredients, the samples were homogenized in an electric shaker (PHILIPS HR3655/00 smoothie maker) for 2 min. Directly after homogenization, the samples were bottled in 250 mL PET bottles and closed.

The heat treatment was carried out in a laboratory water bath at 65°C for 3 h. Both parameters were selected due to the recommendations of the ToTu beverage's producer. Following heat treatment, samples were immediately cooled to 10°C in melting ice. The measurements were carried out 24 h after the heat treatment.

### 2.2. Methods

#### 2.2.1. Determination of rheological properties

The samples after heat treatment and cooling period showed smoothie-like or yogurt beverage-like consistency depending on the mixed berries' concentration, so their rheological properties were analyzed by Anton Paar MCR 92 rheometer (Anton Paar, France) in rotational mode equipped with a concentric cylinder (CC27) similar to Hidas et al. (57). Anton Paar RheoCompass software (v 1.21.852) was used to control the measurements. The temperature of the rheological experiment was kept constant at 15°C. Shear stress was measured in increasing and decreasing shear rate intervals between 10 and 1,000 s^−1^ for 31 measurement points in each interval with a period of 3 s.

The Herschel-Bulkley model (see equation below) was used to analyze the flow curves (shear rate-shear stress diagrams) data of decreasing shear rate interval were analyzed using Excel solver (58–60)


(1) $$\begin{array}{l} \tau =\tau_{0}+K\gamma^{n} \end{array}$$

° *τ*—shear stress (Pa);
° *τ*_0_–yield stress (Pa);
° γ–shear rate (s^−1^);
° K—consistency coefficient (Pa s^n^);
° *n*—flow behavior index (dimensionless).

This model was used to describe the rheological properties of the samples. All determination coefficient values (R^2^) of the fitted models were higher than 0.98.

#### 2.2.2. Determination of total anthocyanin concentration (TA)

In this method, the anthocyanin content of egg white-based beverage samples was measured by the pH differential method presented by Lee et al. (61) and Taghavi et al. (62). One mL of the egg white-based beverage samples was mixed thoroughly with 19 mL buffer pH 1.0 (0.025 M potassium chloride) and 1 mL of the egg white-based beverage samples was mixed thoroughly with 19 mL buffer pH 4.5 (0.4 M sodium acetate buffer). Both sample-buffer solutions were incubated for 20 min at room temperature and centrifuged at room temperature, for 15 min, at 1,0000 rpm (Hettich MIKRO 120, Andreas Hettich GmbH. Tuttlingen, Germany). The supernatant was then removed, and the absorbance was read at 520 and 700 nm (Hitachi U-2900 spectrophotometer). The following equation was used for the calculation of the anthocyanin concentration.


(2) $$\begin{array}{l} TA=A*VM \end{array}$$

- A: (A520 nm–A700 nm) pH 1.0 – (A520 nm–A700 nm) pH 4.5;
- V: volume of extract (mL);
- M: mass of the egg white-based beverage sample (g).

#### 2.2.3. Determination of total antioxidant capacity

The total antioxidant capacity was obtained by FRAP (Ferric Reducing Antioxidant Power) method described by Moure et al. (63). The method reported by Carletti et al. (64) was slightly modified for the measurement of FRAP of the egg white-based beverage samples.

The FRAP reagent was freshly prepared as a mixture of acetate buffer (300 mM, pH = 3.6), TPTZ (10 mM), and ferric chloride (20 mM) at a 10:1:1 (v/v/v) ratio, respectively. Then, 3 mL of the FRAP reagent and 100 μL of the sample were mixed and incubated at 30°C for 30 min in a water bath avoiding light. The absorbance was determined at 593 nm using a Hitachi U-2900 spectrophotometer. Calibration was carried out using ascorbic acid solutions between 0.01 and 0.1-mM concentrations. The results were expressed as g ascorbic acid equivalent/l egg white-based beverage.

#### 2.2.4. Determination of total polyphenols content (TPC)

The total polyphenolic content (TPC) was determined using the colorimetric Folin–Ciocalteu method (65) by spectrophotometric analysis (spectrophotometer Hitachi U-2900). The measurement was carried out similarly to Musilova et al. (66). The absorbance of blue solutions was measured in cuvettes of 1 cm width at a wavelength of 765 nm. The calibration was carried out with gallic acid solutions in a concentration range between 0.01 and 1.5 g/L. The content of total polyphenols in the sample was expressed as the content of gallic acid in g/l of the sample.

#### 2.2.5. Experimental design: Central composite rotational design (CCRD)

For the experiment, a central composite rotational design (CCRD) with two variables was performed for design and data evaluation as described by Box and Draper (67). The two variables were the concentration of mixed berries (factor A) and the bovine collagen peptide concentration (factor B). The factor levels are demonstrated in Table 1. The concentrations of mixed berries and bovine collagen peptides used for every sample are presented in Table 2.

**Table 1:** The trial design and factor levels in encoded values applied in the central composite rotational design (CCRD) (^*^is a theoretically calculated value, 0/100 g bovine collagen peptides were added).

**Variable**	**Encoded factor**	**−1.4142**	**−1**	**0**	**1**	**1.4142**
mixed berries concentration, g/100 g	X1	1.89	5.00	12.50	20.00	23.11
bovine collagen peptides concentration, g/100 g	X2	−0.02^*^	0.50	1.75	3.00	3.52

**Table 2:** The calculated concentrations of mixed berries and bovine collagen peptides according to the central composite rotational design (CCRD) (^*^is a theoretically calculated value, 0/100 g bovine collagen peptides were added).

**CCRD**	**test nr**.	**Mixed berries concentration, g/100 g (A)**	**Bovine collagen peptides concentration, g/100 g (B)**
^*^L:A-a	1	1.89	1.75
^*^H:A-a	2	23.11	1.75
^*^L:B-a	3	12.50	−0.02^*^
*H:B-a	4	12.50	3.52
Cube001a	5	5.00	0.50
Cube002a	6	20.00	0.50
Cube003a	7	5.00	3.00
Cube004a	8	20.00	3.00
Cent-a	9	12.50	1.75
Cent-b	10	12.50	1.75
Cent-c	11	12.50	1.75

For approximation, we used the response surface obtained based on the secondary polynomial model. The measurements were carried out in a random order, and the data were analyzed by specific software (The Unscrambler 10.0.0 Camo-software, Norway). The general form of the polynomial model used in our study is described by the following equation:


(3) $$Y=\beta_{0}+\beta_{1}X_{1}+\beta_{2}X_{2}+\beta_{11}*X_{1}^{2}+\beta_{22}X_{2}^{2}+\beta_{12}X_{1}X_{2},$$

° *Y* = independent variable;
° *β*_1_, *β*_2_, *β*_11_, *β*_22_, *β*_12_ = regression coefficients;
° *X*_1_ = a mixed berries concentration, % m/m;
° *X*_2_ = bovine collagen peptides concentration, % m/m.

Three replicates of the center point were selected (12.5 g/100 g mixed berries and 1.75 g/100 g bovine collagen peptides' concentration) based on a pilot experiment considering the sensorial attributes of enriched and flavored egg white-based beverage. The independent variables (a: mixed berries and B: bovine collagen peptides) were varied between 1.89 and 23.11 and 0.0 and 3.52/100 g, respectively, and dependent variables of *τ*_0_, n K, TA, FRAP, and TPC were measured.

## 3. Results and discussion

### 3.1. Changes in rheological properties

**Figure 1:**
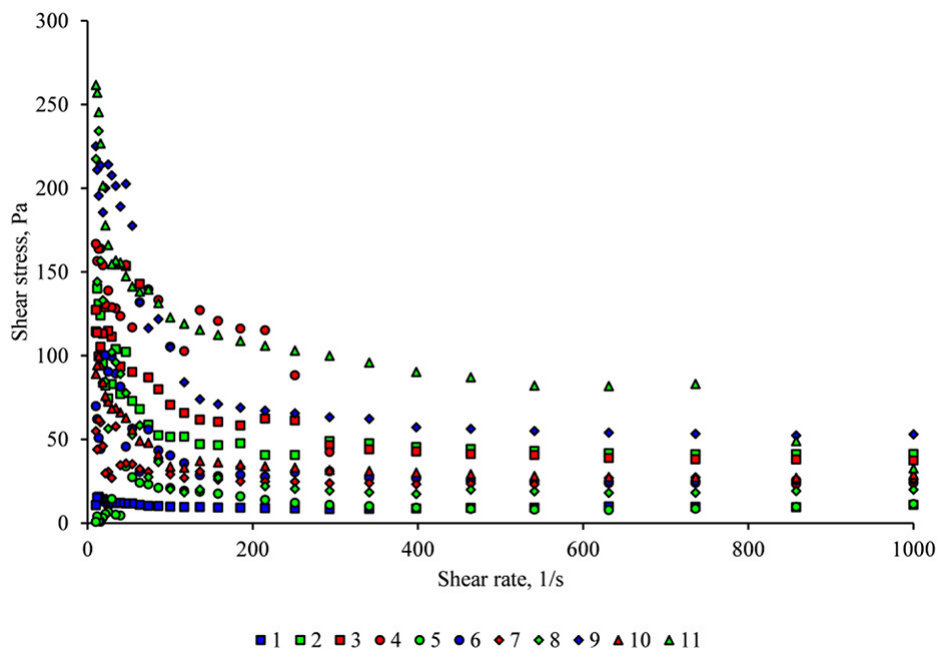
The flow curves (shear stress vs. shear rate) of egg white-based beverages with different mixed berries and bovine collagen peptide concentrations (samples are coded by test numbers).

Figure 1 shows the flow curves (shear stress vs. shear rate), which presents the downward curves of all examined samples. The upwards curves are not shown in this study because it would make the figure crowded. The samples behave as non-Newtonian fluids, namely the pseudoplastic type. According to Holdsworth (68), most fluid foods exhibit pseudoplastic behavior, where the shear stress and the apparent viscosity decrease with increasing deformation rate (22).

However, they may vary highly in rheological behavior due to their complex structure and composition (concentration and ripening stage of fruit ingredients, etc.). Shear stress values of almost all samples show some outlier values between 10 and 100 1/s shear rates, which may be explained on the one hand by the inaccurate measurement at low shear stress values, on the other hand by the slight foaming of samples observed during the rheological investigation.

*τ*_0_ values calculated by the Herschel-Bulkley model varied between 0.12 and 37.38 Pa (Table 3). The lowest *τ*_0_ value was calculated for the sample containing the lowest mixed berry concentration (1.89/100 g, sample 1), and its flow curve shows the lowest shear stress values in the entire investigated interval. The highest *τ*_0_ value (37.38) was calculated for sample 11. On the flow curves, the highest shear stress values are observed for the core points of CCRD (samples 9, 10, and 11) containing 12.5 g/100 g mixed berries and 1.75 g/100 bovine collagen peptides. It might suggest that the mixed berries' concentration (factor A) has a positive and the bovine collagen peptides' concentration has a negative correlation with *τ*_0_ values. Thus, in CCRD, a positive β-coefficient of factor A and a negative β-coefficient for B are calculated. However, the CCRD model is not significant (*p* = 0.529). It means that *τ*_0_ values can't be estimated for the examined beverage samples.

**Table 3:** Experiment design [central composite rotational design (CCRD)] and factor levels with actual values, and measured results of Herschel-Bulkley model and bioactive compounds (^*^is a theoretically calculated value, 0/100 g bovine collagen peptides were added).

**Test nr**.	**Mixed berries concentration, g/100 g (A)**	**Bovine collagen peptides concentration, g/100 g (B)**	***τ*_0_, Pa**	**k, Pa s^n^**	**n, -**	**R^2^**	**Total anthocyanin concentration, mg/L**	**FRA*P*-value, g/L**	**Total polyphenol content, g/L**
1	1.89	1.75	0.12 ± 0.05	0.01 ± 0.004	1.14 ± 0.21	0.99	3.42	0.63	0.05
2	23.11	1.75	21.43 ± 1.31	11.97 ± 0.92	0.60 ± 0.09	0.98	141.97	1.19	1.03
3	12.50	−0.02^*^	36.48 ± 2.47	7.36 ± 0.58	0.65 ± 0.04	0.99	60.27	0.84	0.69
4	12.50	3.52	11.47 ± 1.16	14.60 ± 1.25	0.65 ± 0.05	0.98	58.97	0.94	0.70
5	5.00	0.50	2.86 ± 0.72	2.06 ± 0.05	0.75 ± 0.06	0.98	6.11	0.73	0.07
6	20.00	0.50	17.54 ± 0.95	6.11 ± 0.24	0.61 ± 0.06	0.99	101.63	1.07	0.85
7	5.00	3.00	3.29 ± 0.12	7.10 ± 0.48	0.64 ± 0.05	0.98	5.03	0.77	0.06
8	20.00	3.00	15.28 ± 1.61	16.34 ± 1.27	0.55 ± 0.04	0.98	98.74	1.04	0.87
9	12.50	1.75	7.96 ± 0.91	15.55 ± 2.01	0.67 ± 0.06	0.99	59.20	1.09	0.71
10	12.50	1.75	7.45 ± 0.84	11.90 ± 0.92	0.67 ± 0.05	0.98	58.90	0.98	0.69
11	12.50	1.75	37.38 ± 2.56	7.58 ± 0.55	0.55 ± 0.03	0.98	60.87	1.00	0.71

The highest flow behavior index, n was calculated for sample 1, containing the lowest mixed berries concentration. If *n* > 1, the flow behavior is considered dilatant or shear thickening. Accordingly, sample 1 should have a dilatant behavior (*n* = 1.14), as long as, all other samples could be described as pseudoplastic fluids (calculated n values between 0.55 and 0.75, Table 4). But dilatancy is considered as the behavior showing an increase in viscosity with increasing shear rate. But investigating (Figure 1) it is clear, that every sample has a decreasing shear stress in the function of shear rate. It means that they have pseudoplastic behavior. In our samples, it is recognized that pseudoplasticity represents an irreversible structural breakdown and the decrease in viscosity occurs as a result of molecular alignment that takes place within such a substance (69).

**Table 4:** The regression coefficients of the quadratic polynomial model for response analysis with encoded units.

**Factors**	*τ*_**0**_ **, Pa**		**n, -**		**K, Pas** ^**−***n*****^		**Total anthocyanin**		**FRAP, g/L**		**Total polyphenol**	
							**concentration, mg/L**				**content, g/L**	
	*β* **-coefficient**	***p*** **-value**	*β* **-coefficient**	***p*** **-value**	*β* **-coefficient**	***p*** **-value**	*β* **-coefficient**	***p*** **-value**	*β* **-coefficient**	***p*** **-value**	*β* **-coefficient**	***p*** **-value**
Constant	17.598	0.5299	0.63	0.1787	11.68	0.0007^*^	59.66	0.0001^*^	1.023	0^*^	0.71	0.0001^*^
A	0.947	0.1831	−1.66E-02	0.0363^*^	0.50	0.0113^*^	6.42	0^*^	2.31E-02	0.0003^*^	4.97E-02	0.0002^*^
B	−3.719	0.3584	−1.67E−02	0.6555	2.552	0.0214^*^	−0.58	0.8185	1.44E-02	0.3835	2.93E-03	0.9242
AxB	−0.536	0.9219	8.78E-03	0.8667	1.04	0.385	−0.36	0.9192	−1.31E-02	0.5677	5.78E-03	0.8946
AxA	−4.256	0.3757	7.04E-02	0.1536	−2.39	0.0481^*^	2.56	0.4117	−4.45E-02	0.0562	−9.61E-02	0.0401^*^
BxB	1.025	0.8242	−1.69E-02	0.7034	−0.39	0.6846	−2.67	0.3933	−5.32E-02	0.0315^*^	−3.58E-02	0.3518

A: Mixed berries concentration, g/100 g; B: Bovine collagen peptides concentration, g/100 g;

* significant effect (*p* < 0.05).

The mixed berries concentration had a significant effect on n values (*p* = 0.0363). Although it showed a negative correlation with both factors, but a positive correlation with their interaction. Despite this, the CCRD model was not significant (*p* = 0.179). Similar flow behavior index values and a negative correlation of fruit concentration flow behavior index were found by Wang et al. (70) in strawberry-flavored goat milk beverages. In aspects of *τ*_0_ and n the applied CCRD statistical model fails in the prediction of the rheological behavior of the beverages. Thus, it can be seen that increasing mixed berries and bovine collagen peptide concentrations have practical effects on rheological properties and have to be considered for further product and technology development.

The calculated values of the Herschel-Bulkley model are presented in Table 3.

**Figure 2:**
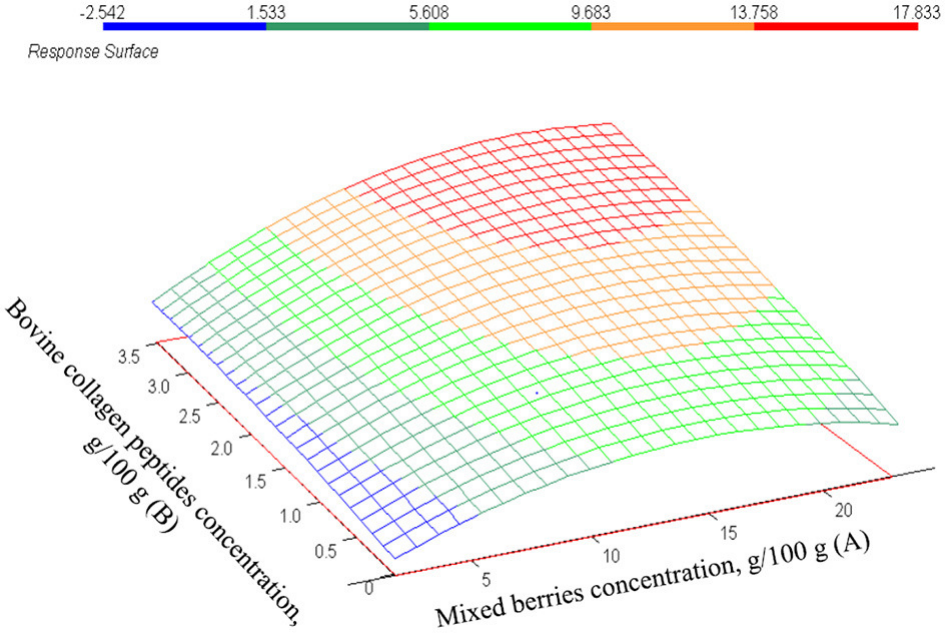
The estimated values of K in function of mixed berries concentration (A) and bovine collagen peptides concentration (B).

**Figure 3:**
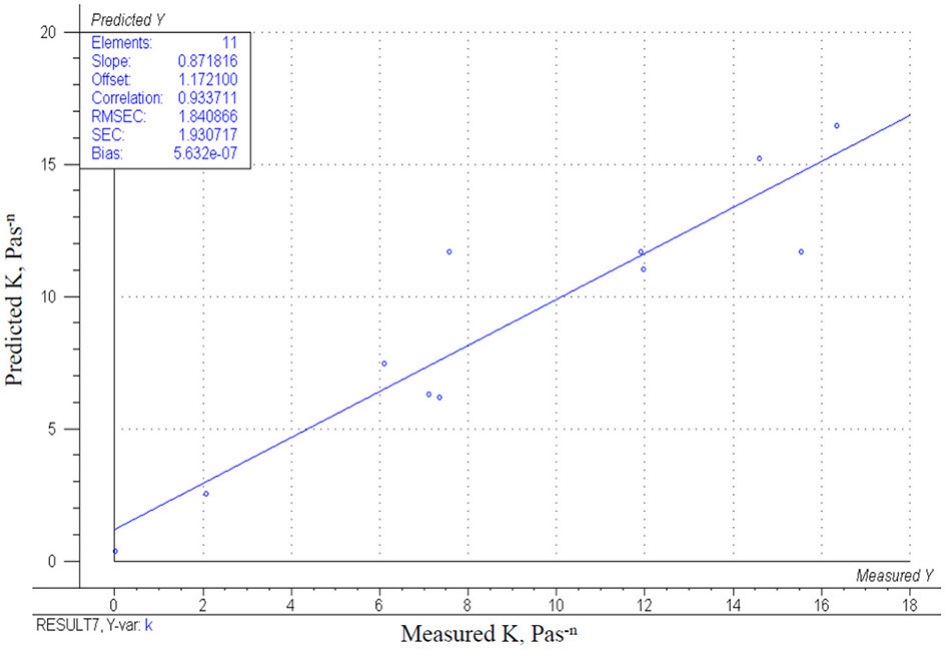
Correlation of the obtained model and the measured results for the change in K.

In the investigation of rheological parameters, only the consistency coefficient (or consistency indices) is described by a statistically significant CCRD model (*p* = 0.0007). The measured K values are summarized in Table 4: their values ranged from 0.01 to 16.34 Pas^n^. The measured K values may imply that the higher mixed berries concentration and/or higher bovine collagen peptides' concentration was used, the higher K values are calculated. This suspension is proven by the *β*-values shown in Table 4. The interaction of both parameters gives a positive β-coefficient. The effects of parameters are demonstrated in Figure 2. The higher consistency coefficient (K) was estimated if higher concentrations of mixed berries or bovine collagen peptides were used. It means that the increase of one or both factors results in a thickener, more contentious beverage. The correlation of the model with the measuring points (*r*^2^ = 0.87) is shown in Figure 3.

Similar results were reported by Penna et al. (71) analyzing the rheological parameters of commercial lactic beverages obtained by the Herschel–Bulkley model. The authors explained their findings by inter- and intramolecular changes in their samples, which may be the reason in our experiment as well.

However, our samples were less consistent than some milk-based beverages reported by Dogaru et al. (72), the difference may be based on the different sweetener types and fruit concentrations used. In practice, the increase of both factors indicated a thicker consistency and more deviation from Newtonian flow behavior, providing a low-fat yogurt beverage with similar flow behavior (73) that could be accepted by consumers as a dairy beverage replacement.

### 3.2. Total anthocyanin concentration

The measured total anthocyanin concentration (TA) of samples is summarized in Table 3. which shows a proportional tendency between 3.42 and 141.97 mg/L in the function of mixed berries concentration. The higher the concentration of mixed berries concentration added, the darker and more reddish-blueish coloration was observed in the samples. This phenomenon seems to be in correlation with TA concentration. For example, the dark blue coloration of blueberries is a result of the high level of anthocyanin concentration (56, 74).

**Figure 4:**
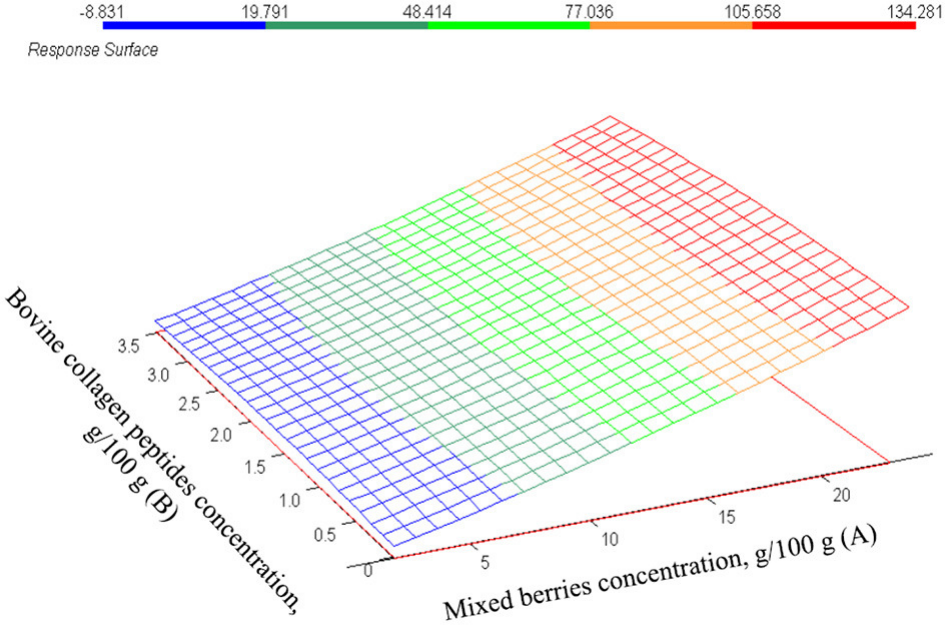
The estimated values of total anthocyanin concentration. mg/L in function of mixed berries concentration (A) and bovine collagen peptides concentration (B).

**Figure 5:**
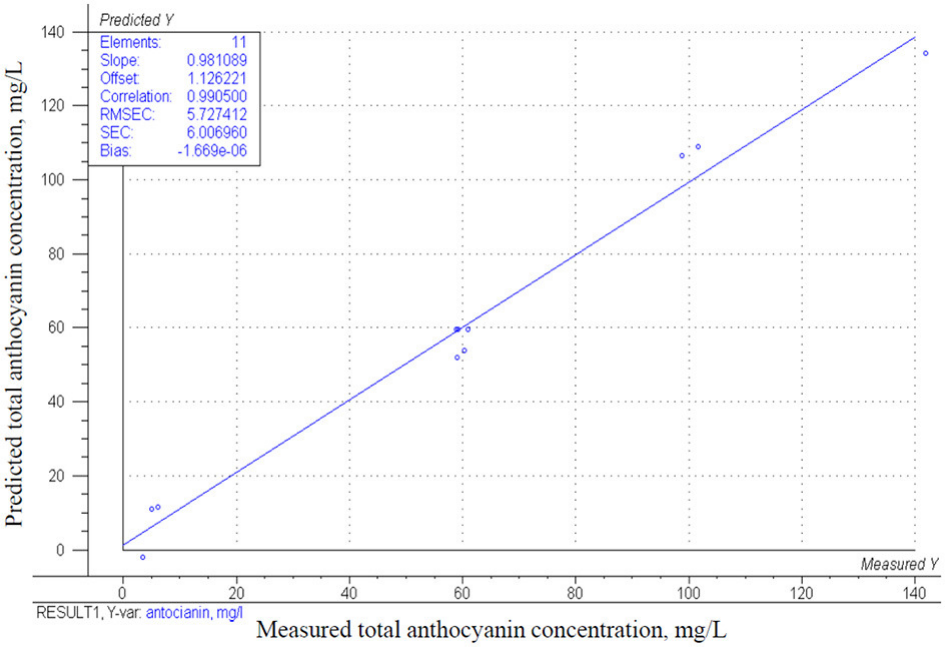
Correlation of the obtained model and the measured results for the change in total anthocyanin concentration mg/L.

Regarding the CCRD model (*p* = 0.0001) the total anthocyanin concentration is significantly influenced only by mixed berries concentration (*p* = 0.000) as presented in Table 4. However, the bovine collagen peptides' concentration has a positive correlation, and the interaction of factors has a negative effect on the total anthocyanin concentration, it is not statistically significant. The estimated effects are represented in the response surface shown in Figure 4. The increasing values of total anthocyanin concentration are clearly visualized as the effect of increasing mixed berries concentration The correlation between the estimated model and measured values is demonstrated in Figure 5 (*r*^2^ = 0.98).

The same order of magnitudes TA were reported in fresh, frozen, and frozen-dried strawberries using a similar method to Taghavi et al. (62). It seems that anthocyanin and phenolic compounds stay stable during cold storage of fruit yogurts due to the bounds between proteins and phenolic compounds (75), the intermolecular actions between bovine collagen, egg white proteins, and anthocyanins haven't been deeply analyzed yet. Although, it can be suggested that collagen peptides can interact with anthocyanin and phenolic compounds (26, 27, 32) and in practice, it may play a key role in the development of long-term stabilized color of egg white-based fruit flavored beverages. From the rheological investigation, it may be concluded that in our samples, intermolecular reactions took place which may help stabilize their color during longer storage as well.

### 3.3. Total antioxidant capacity

Table 3 presents the measured values of total antioxidant capacity expressed in g/l ascorbic acid equivalent FRAP values. The measured antioxidant capacities (0.63 1.9 g/l) meet the range of published values found in berries (74) and reported in fruit-flavored yogurts (76). Antioxidant concentrations of fresh blueberries and strawberries were reported at 1,107.14 and 199.49 mg/kg, respectively by Mustafa et al. (77) using an HPLC-MS/MS method. However, the method used in our study is different, the magnitude of measured FRAP values are showing a similar antioxidant concentration.

**Figure 6:**
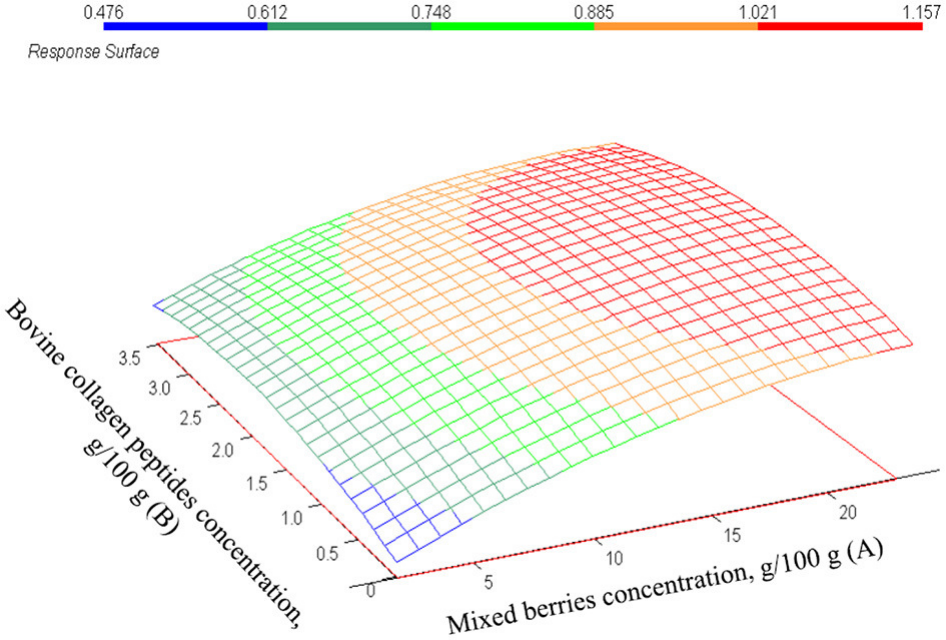
The estimated values of FRAP g/L in function of mixed berries concentration (A) and bovine collagen peptides concentration (B).

**Figure 7:**
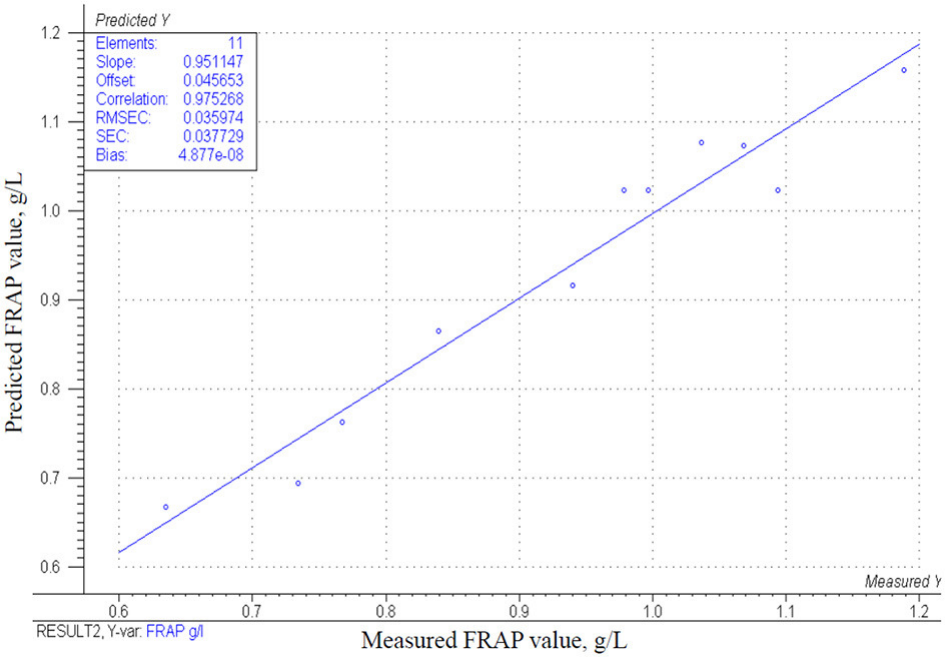
Correlation of the obtained model and the measured results for the change in FRAP g/L.

The data of the fitted CCRD model are summarized in Table 4: the model is significant (*p* = 0.000). Similar to the total anthocyanin concentration, mixed berries concentration has a significant effect on the FRAP values (*p* = 0.0003) and its correlation is positive to the measured values. However, collagen peptides have antioxidant activity (49, 51), and show a positive correlation (β-coefficient = 1.44E-02) in our model, their effect is not statistically significant (*p* = 0.383). The estimated values of total antioxidant capacity are shown in Figure 6 as a function of the bovine collagen peptides and mixed berries concentrations. If the goal is to achieve the highest total antioxidant capacity in egg white-based berry flavored beverages, a wide range of bovine collagen peptides' concentration (between 0.5 and 3.2/100 g) and a higher mixed berries concentration (between 28 and 23/100 g) are recommended. Figure 7 represents the correlation (*r*^2^ = 0.95) of measured and estimated FRAP values.

A preservation technology applied for extending shelf-life is required for all food products. In the case of milk and egg products, heat treatments are commonly applied. The heat treatment seems to increase antioxidant capacity and bioavailability of bioactive compounds (53, 66), so in this context, the heat treatment applied in our experiment may improve the bioactivity of the egg white-based beverages.

### 3.4. Total polyphenols content

**Figure 8:**
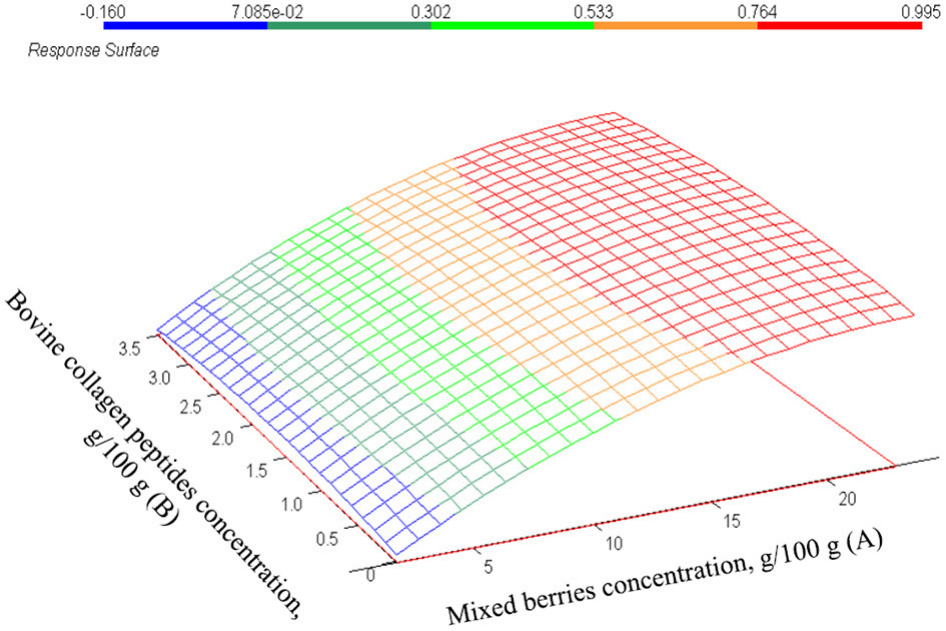
The estimated values of total polyphenol content. g/L in function of mixed berries concentration (A) and bovine collagen peptides concentration (B).

**Figure 9:**
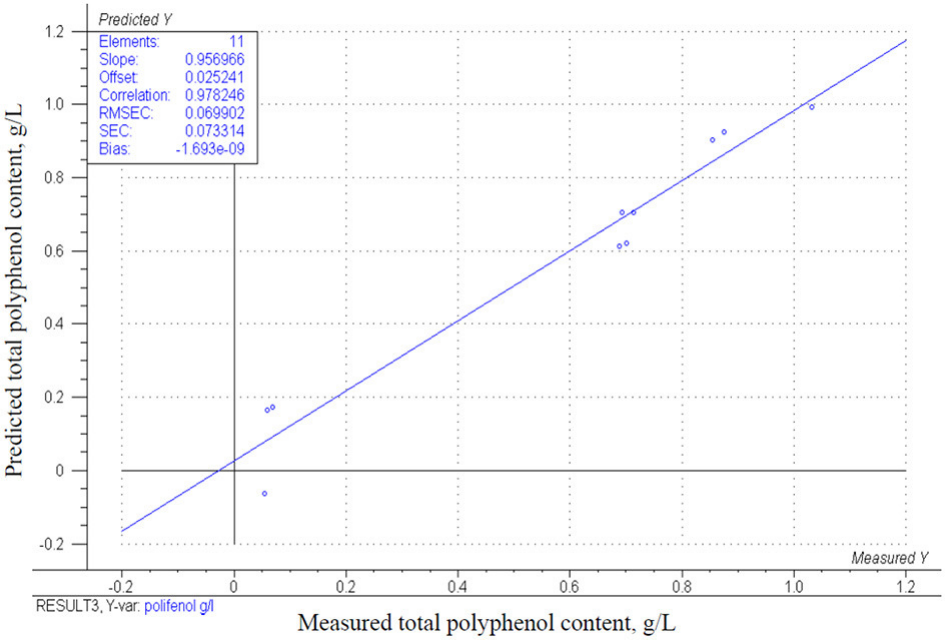
Correlation of the obtained model and the measured results for the change in total polyphenol content g/L.

The measured total polyphenol concentrations (TPC) presented in Table 3 vary greatly between 0.05 and 1.03 g/L. A positive correlation between mixed berries concentration and TPC might be assumed from measured values. This finding is supported by the results of the estimated CCRD model (*p* = 0.0001) described in Table 4. Regarding the regression coefficients and *p*-values, the mixed berries concentration has a significant effect (*p* = 0.0002) on TPC and the correlation of both factors are positive (β-coefficient = 4.97E-02; β-coefficient = 2.93E-03, respectively) as well as their interaction correlates positively (β-coefficient = 5.78E-03), although factor B and the interaction of both factors are not significant (*p* = 0.924 and *p* = 0.895, respectively). This is shown in Figure 8. The estimated values and measured values are demonstrated in Figure 9 (*r*^2^ = 0.96).

Blueberry is considered a berry fruit rich in phenolic compounds, ascorbic acid, and other antioxidants (78) as discussed above. TPC values were reported in a range of 77–82 mg/100 g for blueberries grown in the Black Sea Region of Turkey (74) and 305.38 ± 5.09 mg/100 g for blueberries from the subtropical areas of Brazil (79). In fresh and frozen raspberries a total phenolic concentration of 185.57 mg GAE/100 g and 1,476.62 mg GAE /100 g were reported, respectively (80). These levels correspond to yogurt beverages enriched with different berries (76) and with our measured results (taking into consideration the used concentrations of mixed berries). However, in most studies investigating the effects of thermal treatments on the antioxidant capacity and total phenolic content, the results are contradictory (81). It seems that some antioxidants and phenolic compounds' concentration increase above 70°C (82). This temperature range for heat treatment is considered to have inactivating and decreasing effects on food spoilage microbiota.

## 4. Conclusions

Modern food consumers, who exclude dairy from their diet or want to make their nutrition more varied, can be successfully encouraged by foods providing high bioactivity and great amino acid composition. To fulfill these requirements, in our study, an egg white-based berry-flavored and collagen peptide-enriched dairy replacement was investigated. For this purpose, a central composite rotational design (CCRD) was used with two factors (mixed berries concentration and bovine collagen peptides' concentration, 1.89–23.11/100 g and 0.00 and 3.52/100 g, respectively). The effects of both factors were examined on modeled rheological parameters (*τ*_0_, K, and n) and bioactive composition (total anthocyanin concentration, antioxidant capacity, and total polyphenolic content) by using response surface methodology.

Berries are used as a well-known source of bioactive compounds and are popular because of their sensorial attributes. They showed positive correlations and significant effects on the examined parameters. Thus, their use in higher concentrations in egg white-based beverages could provide a high quantity of bioactive compounds for consumers. Nevertheless, their sensorial characteristics (color, flavor, and taste formation) effects are popular among consumers, regardless of gender and age. For further investigations and product development, mixed berries concentrations of 12.5/100 g and 23.11/100 g (or even higher) are recommended to increase the concentration of bioactive compounds and to achieve superior sensorial attributes.

Despite this, bovine collagen peptides, which are rich in highly bioactive and digestible amino acids, had lower effects on the investigated parameters, despite this, according to the literature, their addition may have a positive effect on the retardation and expression of bioactive compounds Their use in dairy replacement products such as yogurt beverage replacements, might be a useful tool for providing higher bioactivity, and higher protein concentration, and a better amino acid composition.

The product development based on the central composite rotatable design presented and investigated in our study obtained potential opportunities to replace traditional dairy beverages with an egg white-based option. Egg white and egg white-based products could be considered cheap and environmentally friendly raw materials for further dairy analogs development, providing a higher functionality through their amino acid composition.

## Data availability statement

The raw data supporting the conclusions of this article will be made available by the authors, without undue reservation.

## Author contributions

AV-T contributed to the conception. AV-T and ID designed the analysis. ID carried out the statistical analysis. AV-T and RC collected data and performed the analysis. AV-T and TC wrote the first draft of the manuscript, and CN and KH contributed to manuscript revision, read, and approved the submitted version. MEl and MEn created Tables and Figures and supervised the manuscript in nutritional aspects. LF revised the draft and supervised the entire work procedure. All authors contributed to the article and approved the submitted version.
